# Robotic Vessel Sealer vs. Robotic Bipolar Grasper in Partial Nephrectomy

**DOI:** 10.3390/cancers18050802

**Published:** 2026-03-02

**Authors:** Murad Asali, Osman Hallak, Galeb Asali

**Affiliations:** 1 Urology Department, Barzilai University Medical Center, Ben Gurion University of the Negev, Beer Sheva 8430905, Israel; osmanh@bmc.gov.il; 2 Assuta Medical Center, Beer Sheva, Ramat Hyal, Ben Gurion University of the Negev, Beer Sheva 8489507, Israel; 3 Faculty of Medicine in Safed, Bar-Ilan University, Ramat-Gan 1311502, Israel; galeb.asali@live.biu.ac.il

**Keywords:** robotic vessel sealer, bipolar energy, partial nephrectomy, ischemia time, nephron-sparing surgery

## Abstract

Background: Robotic-assisted partial nephrectomy (RAPN) is widely used for nephron-sparing surgery. Vessel sealing technologies play a crucial role in these procedures, influencing ischemia time, blood loss, and surgical outcomes. This study compares the efficacy and safety of using a robotic vessel sealer (VS) versus conventional bipolar energy in robotic partial nephrectomy. Methods: A retrospective analysis was conducted on patients (n = 112) undergoing RAPN using either a robotic VS or bipolar energy grasper between 2023 and 2025. Parameters analyzed included ischemia time, estimated blood loss (EBL), postoperative complications, and functional outcomes. Statistical comparisons were performed to assess differences in perioperative and postoperative metrics. Results: The vessel sealer (VS) group (n = 54) had significantly lower blood loss (40 mL vs. 132.5 mL, *p* = 0.037) than the bipolar group (n = 58). Ischemia time was similar (24.5 min vs. 20.46 min, *p* = 0.444). No significant differences were found in operative time, console time, docking time, or postoperative complications (*p* > 0.05). The tumor diameter (CT: 28.38 mm vs. 25.5 mm, *p* = 0.655; pathology: 2.34 cm vs. 1.96 cm, *p* = 0.375) and Radius–Exophytic/Endophytic–Nearness to collecting system or sinus–Anterior/posterior–Location relative to polar lines (RENAL) score (7.00 vs. 6.17, *p* = 0.202) were slightly higher in the bipolar group but not statistically significant. Conclusions: Both techniques seem to be used for tumors of comparable size and complexity, suggesting no strong selection bias. Future research should prioritize randomized controlled trials assessing long-term renal function, cost-effectiveness, and potential refinements of robotic vessel sealing technology. A broader, multicenter analysis could provide further insight into optimal device selection for robotic partial nephrectomy.

## Simple Summary

This single-center study evaluates two energy devices used during robotic-assisted partial nephrectomy (RAPN): a robotic vessel sealer (VS) and a conventional robotic bipolar grasper. A total of 112 patients who underwent RAPN between 2023 and 2025 were retrospectively analyzed, with perioperative, oncologic, and postoperative outcomes compared between groups. Tumor size, RENAL nephrometry score, operative time, ischemia time, console time, and complication rates were similar, indicating comparable case complexity and no evident selection bias. The key finding was a statistically significant reduction in estimated blood loss in the VS group compared with the bipolar group, without differences in ischemia time or postoperative morbidity. Both devices demonstrated safety and effectiveness, but the VS provided superior hemostasis without improving overall operative efficiency. The authors conclude that while robotic VS may be advantageous in cases where minimizing blood loss is critical, bipolar graspers remain a reliable and cost-effective alternative. Larger multicenter and randomized studies are needed to assess long-term renal function and cost-effectiveness.

## 1. Introduction

Minimally invasive surgery has revolutionized the field of urology, with robotic-assisted techniques playing a pivotal role in complex renal surgeries such as partial nephrectomy [1,2]. Robotic-assisted partial nephrectomy (RAPN) has lower minor complication rates, with potential advantages in warm ischemia time and complication rates compared with laparoscopic partial nephrectomy (LPN) [3]. Based on what Bao et al. published, there is an advantage to RAPN compared to LPN; they recommended RAPN as the first-line treatment for hilar tumors [4]. RAPN may facilitate earlier recovery of ipsilateral GFR compared to open partial nephrectomy [5].

Two commonly used energy devices in robotic-assisted surgery are the robotic vessel sealer (VS) and the robotic bipolar grasper, both of which are utilized to achieve hemostasis and tissue dissection [6,7].

The robotic bipolar grasper operates by delivering controlled, high-frequency electrical energy to coagulate tissues and seal blood vessels. This technique has been widely adopted due to its reliability in coagulation and precise energy delivery, making it an effective tool for minimizing blood loss. However, its sealing efficiency for larger vessels remains a limitation, often requiring additional suturing or hemostatic agents. In contrast, the bipolar VS offers the efficacy of bipolar diathermy and the advantages of a fully wristed instrument. It does not require any change in instruments for coagulation or involvement of the bedside assistant surgeon. These characteristics lead to a reduction in operative time [8,9]. Moreover, the VS is an advanced energy device designed to simultaneously seal and transect vessels up to a certain diameter without requiring additional sutures or clips. By utilizing a combination of bipolar energy and mechanical pressure, the vessel sealer achieves a secure and uniform seal, which may reduce intraoperative bleeding and ischemia time, potentially leading to improved surgical efficiency and outcomes [7].

Although no study has directly compared these devices in robotic partial nephrectomy, Kong et al. showed that VS in robotic gastrectomy was feasible and provided good configuration in the direction of dissection. The learning process for the use of the VS in the initial series was relatively rapid, resulting in comparable results between the VS and ultrasonic groups. Reduced inflammation and albumin loss were identified as possible benefits of the VS.

This study aims to compare the effectiveness of these two energy devices in robotic-assisted partial nephrectomy, focusing on key surgical and clinical outcomes.

## 2. Methods and Patients

This study is a retrospective and prospective analysis of patients undergoing RAPN at a single center between 2023 and 2025. The inclusion criteria encompassed patients with localized renal masses amenable to nephron-sparing surgery. Patients were divided (Table 1) into two cohorts based on the energy device used: robotic vessel sealer (Group 1) and robotic bipolar grasper (Group 2).

### 2.1. Data Collection

- Patient demographics (age, sex, BMI, comorbidities, tumor characteristics).
- Perioperative outcomes (operative time, console time, docking time, ischemia time, estimated blood loss (EBL)).
- Postoperative recovery (hospital stay, complications).

Statistical Analysis: Comparative analysis was performed using Student’s *t*-test and chi-square test for continuous and categorical variables, respectively. A *p*-value < 0.05 was considered statistically significant.

The Clavien–Dindo classification was used to report complications [10].

All procedures were performed by a single senior robotic surgeon (M.A.) with extensive experience, assisted by the same dedicated robotic team. This minimizes inter-operator variability and strengthens internal validity.

### 2.2. Surgery Technique

All cases were performed using a transperitoneal approach, ensuring uniformity between groups. Since both cohorts were managed identically, this does not introduce intergroup bias. Continuous water irrigation was not used during tumor resection. Standard suction was applied when necessary. Pneumoperitoneum pressure was maintained at a constant level (15 mmHg). This ensures that the estimated blood loss (EBL) measurement was not artificially altered by irrigation fluid.

In all cases, patients were positioned in the lateral decubitus position. Three robotic arms were used—one for the camera and two for the working instruments—along with additional assistant trocars, two on the left side and three on the right side, including one for liver retraction.

Ischemia time was measured starting from the moment of renal artery clamping.

In all cases, an excision technique was performed rather than enucleation or any other combined approach.

The use of VS versus bipolar instruments involved one device per procedure. The instrument was provided by the operating room staff based on availability, without any specific request or preference from the surgeon.

## 3. Results

As shown in Table 2 and Table 3, perioperative outcomes between the robotic vessel sealer (VS) and bipolar grasper groups were largely comparable. Mean ischemia time was slightly shorter in the bipolar group (20.46 vs. 24.5 min), though not statistically significant (*p* = 0.444). In contrast, estimated blood loss was significantly lower with the VS (40.0 vs. 132.53 mL, *p* = 0.037), indicating superior hemostatic performance. Total operative time was nearly identical between groups (169.83 vs. 166.86 min, *p* = 0.654). Console time (113.83 vs. 127.88 min, *p* = 0.253) and docking time (14.67 vs. 18.95 min, *p* = 0.245) were slightly shorter with the VS, but differences were not significant. No conversions to open surgery were recorded, and clamping techniques were similar between groups. Postoperative complications were comparable (*p* = 1.0). Only one minor Clavien–Dindo I complication occurred in the bipolar cohort. Tumor size, Radius–Exophytic/Endophytic–Nearness to collecting system or sinus–Anterior/posterior–Location relative to polar lines (RENAL) score, and hospital stay did not differ significantly.

There were no positive surgical margins in the VS group, while in the bipolar group, there were two cases in which the tumor reached the inked margins.

Overall, both devices demonstrated safety and efficacy, but the VS achieved a clear advantage in minimizing intraoperative blood loss without compromising operative efficiency or recovery.

## 4. Discussion

The findings of this study suggest that both the robotic vessel sealer and robotic bipolar grasper are effective for hemostasis during robotic-assisted partial nephrectomy, but the vessel sealer may provide some additional advantages. Knowing that ischemia time is of great importance, numerous studies have suggested that reducing ischemia time in partial nephrectomy is critical for preserving long-term renal function [11,12,13]. Our study demonstrates that while the VS significantly reduces blood loss (*p* = 0.037), it does not significantly decrease ischemia time (*p* = 0.444). This highlights a trade-off between improved hemostasis and ischemia control, requiring further evaluation in future studies.

Previous research has demonstrated that vessel sealing technology improves hemostasis and reduces intraoperative blood loss in robotic surgeries [14]. There are no articles comparing these instruments in robotic partial nephrectomy; however, the effect of the da Vinci VS on robot-assisted laparoscopic prostatectomy (RALP) complications was studied by Pellegrino et al. who concluded that the use of the VS was significantly associated with a very small reduction in blood loss (22 cc) but with a 32 min increase in the operating room time. They claimed that the VS would have, at best, a very small effect on RALP outcomes, which is of highly questionable relevance given its cost [14].

While the VS has advantages in hemostasis and learning curve, cost considerations may influence device selection [15,16].

The robotic bipolar grasper remains a cost-effective option with reliable coagulation capabilities. Delto et al. suggested that although VSs may improve efficiency, the cost–benefit ratio must be carefully considered. They concluded that minor changes to surgeon preference and creative utilization of instruments in robotic radical prostatectomy can eliminate 40% of costs incurred on robotic instruments alone. Moreover, EBL, operative times, and intraoperative complications are not compromised as a result of cost reduction [17].

Therefore, cost-effectiveness analyses are warranted to assess the economic feasibility of widespread vessel sealer adoption in robotic surgery. While VSs may offer improved efficiency, their higher initial costs compared to bipolar graspers must be balanced against potential benefits such as reduced hospital stays and lower transfusion rates.

The approximate cost of the devices at our institution is as follows:

- Robotic vessel sealer: approximately 800 USD per case (single-use instrument allocation).
- Bipolar grasper: reusable and significantly lower per-case cost (5700 USD/10 cases = 570 USD per case).

It is noteworthy that the robotic VS, an advanced device capable of simultaneous dissection, sealing, and cutting, did not result in a shorter operative time or reduced ischemia time. We think that there are several possible reasons for this discrepancy.

First, the robotic bipolar grasper tip is equipped with a fine, narrow tip that allows for delicate tissue manipulation and precise hemostasis. This slender design provides better access to confined surgical spaces, such as the renal hilum, where meticulous dissection around major vessels and the ureter is required. The narrow tip also enables surgeons to perform fine tissue separation while minimizing the risk of thermal damage to adjacent structures.

One limitation of the bipolar grasper, however, is its reduced sealing capability for larger vessels. Although effective for small to medium-sized vessels, it often requires additional hemostatic techniques, such as suturing or hemostatic agents, when used in highly vascularized areas [18].

Electrocoagulation diathermy is not dependable for vessels that are larger than 2 mm in diameter [19]. Thermal energy is disseminated between the closed branches of the instrument, enabling devices with bipolar energy to seal vessels with a diameter of up to 7 mm. The likelihood of lateral thermal propagation is diminished [20]. A novel method of enhancing the properties of diathermic hemostasis is the use of a hemostatic system that is based on a combination of bipolar electrical energy, high mechanical and low voltage pressure, and the ability to seal vessels with a diameter of up to 7 mm using high-current electricity [21,22]. VS is a device that has been specifically engineered for the da Vinci system. It is equipped with advanced bipolar energy, a cutting function, and a completely wristed function to facilitate more precise movements [8].

**Figure 1:**
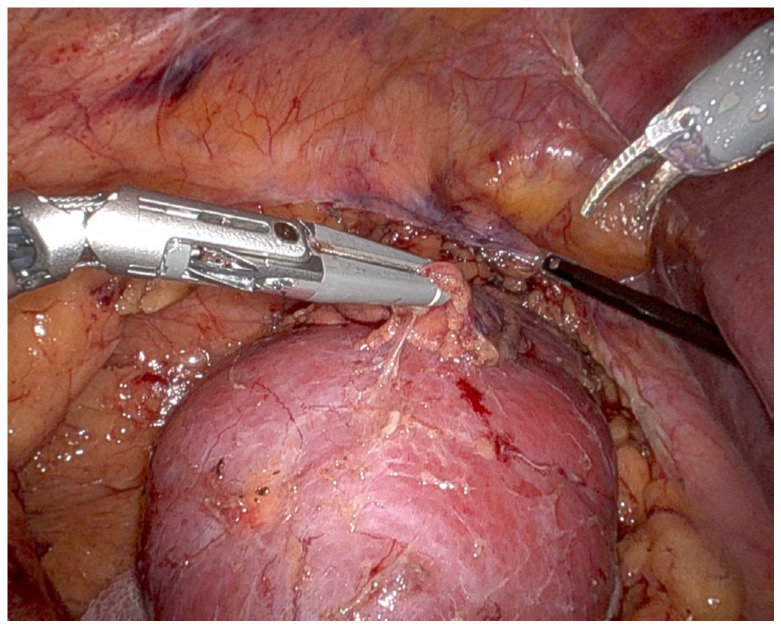
Robotic vessel sealer.

**Figure 2:**
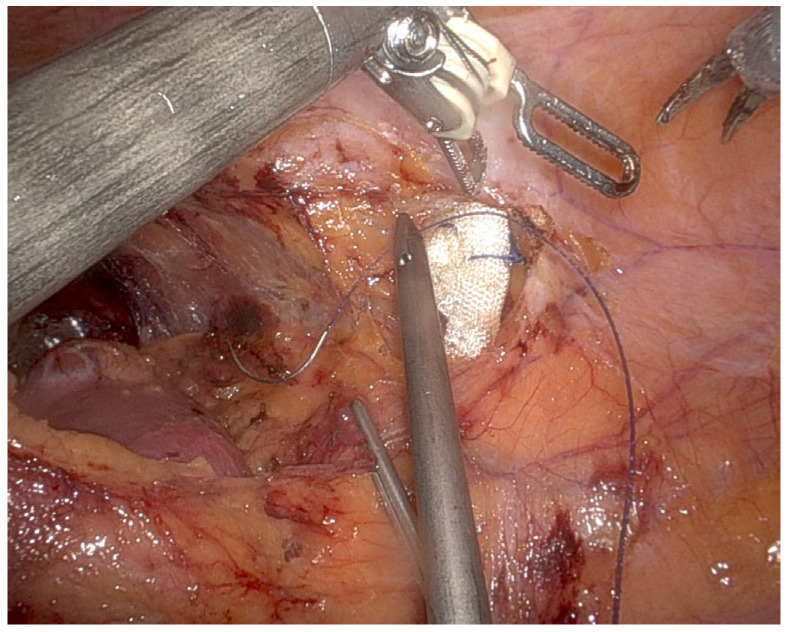
Robotic bipolar grasper.

When examining the robotic VS tip (Figure 1 and Figure 2), it is easy to see that it is a wider, bulkier tip because it is designed to integrate both bipolar energy and mechanical compression for enhanced vessel sealing. While this configuration provides superior hemostasis and efficient transection of larger vessels, its larger tip size can pose challenges in tight anatomical regions. The thick tip of the VS may limit maneuverability in confined spaces, particularly in hilar dissection, where precision is required to avoid injury to the renal artery, renal vein, and surrounding structures. When dissecting near the tumor, where controlled movement and precise margin control are critical, the bulkier tip may reduce the surgeon’s ability to perform fine, controlled movements. This is especially relevant in cases of complex renal tumors with irregular borders or deep hilar involvement.

Despite these challenges, we showed that VS reduced blood loss but did not decrease the operating time or the console time. Increased bleeding with bipolar forceps may also occur, particularly during:

- Hilar dissection when sealing small arterial branches.
- Dissection near sinus fat or endophytic components.

The bipolar grasper provides precise coagulation but has limited sealing capability for vessels > 2–3 mm, sometimes requiring repeated activation or adjunctive suturing. In contrast, the vessel sealer provides mechanical compression plus bipolar energy, creating more consistent sealing of medium-caliber vessels, which likely explains the reduced EBL observed in our cohort.

Second, the sealing time appears to be longer than that of the bipolar grasper, potentially adding extra seconds each time the device’s jaws are activated.

Third, an important point is the insertion of the bulldog clamp for vascular occlusion. The tip of the bipolar device is excellent for grasping, maneuvering, and guiding the bulldog clamp to the renal hilum without trauma, while providing an excellent three-dimensional view. This is not possible when using the VS, requiring the surgeon to delegate the task to the assistant, who lacks a three-dimensional view and whose instruments do not have sufficient articulation. As a result, valuable time is lost in attempts to insert or reposition the bulldog clamp.

Tissue dissection plays a critical role in RAPN, as it determines the extent of nephron preservation, oncologic safety, and procedural efficiency. The precision of dissection directly affects postoperative renal function, surgical margin status, and intraoperative complications. The use of LigaSure has become widely adopted in laparoscopic partial nephrectomy, and it has been proven that the minimal additional tissue excised does not affect renal function and is insignificant compared to sharp dissection with scissors [23]; however, our study shows that the situation is not similar when using the robotic (LigaSure) VS.

Recent multi-specialty data help contextualize our findings on blood loss and operative efficiency when using robotic vessel sealers (VSs) versus conventional bipolar forceps. In hepatobiliary surgery, a large comparative series of robotic liver resections reported on SynchroSeal (an articulating advanced bipolar device) versus the da Vinci vessel sealer, finding both approaches feasible for parenchymal transection, with perioperative outcomes driven more by case complexity than device choice—supporting the notion that energy platform alone rarely determines global efficiency metrics in complex robotics [24,25]. In gynecologic oncology, a single-center series evaluating para-aortic lymphadenectomy with or without the VS on the da Vinci X platform showed that VS integration was safe and effective, facilitating dissection in challenging planes—evidence that aligns with our observation of reliable hemostasis with VS during hilar and parenchymal work [26].

Beyond feasibility, meta-analytic data suggest that advanced bipolar vessel sealing devices can modestly reduce intraoperative blood loss and operative time versus conventional bipolar in laparoscopic hysterectomy; however, the authors judged the absolute differences to be of limited clinical impact—an important caveat when interpreting statistically significant but small effect sizes [27]. Reduced-port gastric cancer surgery has likewise adopted articulating VS devices: in a 2025 series of reduced-port robotic distal gastrectomy, the vessel sealer extend (VSE) supported efficient lymphadenectomy across constrained angles, reinforcing the ergonomic advantage of wristed bipolar sealers in tight fields—anatomic realities that are analogous to renal hilum work in RAPN [28]. The bipolar energy device (SynchroSeal) reported favorable feasibility and safety profiles and referenced earlier experience with the first-generation articulating VS; together, these data indicate that intraoperative improvements (jaw design, articulation, generator algorithms) may incrementally enhance sealing speed and consistency without compromising safety [28]. From a technology standpoint, cross-disciplinary reviews of energy in robotic surgery emphasize that advanced bipolar platforms provide strong burst pressures with limited lateral thermal spread, while real-time impedance-based control helps standardize seal quality—mechanisms that plausibly underlie our observed reduction in blood loss with VS even when ischemia time and global operative time do not improve [29,30]. Notably, early perioperative endpoints reflect only part of the value proposition; contemporary series in non-urologic robotics increasingly evaluate inflammatory markers (e.g., CRP, albumin) and recovery metrics when comparing articulating bipolar sealers to ultrasonic systems, suggesting future RAPN studies should incorporate similar biologic surrogates and patient-centered outcomes to capture benefits beyond minutes saved [28,30].

Taken together, 2020–2025 evidence across liver, gastric, and gynecologic robotics indicates that articulating bipolar sealers (including VS/VSE and SynchroSeal) consistently deliver dependable hemostasis and ergonomic access, with modest and procedure-dependent gains in efficiency. These findings mirror our RAPN experience: VS significantly lowered blood loss yet did not shorten ischemia or console times. The literature also cautions that absolute effect sizes can be small, underscoring the need for adequately powered, RAPN-specific randomized or multicenter studies that (i) stratify by tumor complexity (RENAL score), (ii) standardize clamp/renorrhaphy strategies, (iii) track cost–utility, and (iv) include biochemical and recovery endpoints. Such designs will clarify whether next-generation bipolar sealers meaningfully improve clinically relevant outcomes in nephron-sparing surgery.

This article is the first of its kind to discuss RAPN while comparing robotic VS and bipolar grasper. It can help us choose the right, and possibly more cost-effective, device without compromising oncological principles, causing excessive bleeding, or requiring conversion to open surgery, while also maintaining reasonable operative and console times.

There is a possibility that new robotic VS devices may be developed from da Vinci or other companies, featuring a finer tip with better articulation, enabling faster sealing and possibly more cost-effective. Such advancements could enhance our ability to perform robotic partial nephrectomy more efficiently, allowing us to benefit from all the advantages.

Future research should focus on randomized controlled trials comparing long-term renal function outcomes. Furthermore, larger multicenter studies could help refine selection criteria for each energy device in different patient populations and explore novel technological advancements to further refine robotic-assisted dissection methods.

### Limitations

Unfortunately, Mayo Adhesion Probability (MAP) scores and the presence of perinephric fat adhesions, which may potentially increase bleeding, were not systematically recorded in our retrospective dataset.

## 5. Clinical Implications

- Surgeons may opt for the VS in cases requiring minimal blood loss, particularly in patients at risk for excessive intraoperative bleeding.
- The bipolar grasper remains a reliable and cost-effective tool with proven efficacy, making it a viable alternative when vessel sealing technology is unavailable.

## 6. Conclusions

Both techniques seem to be used for renal tumors of comparable size and complexity, suggesting no strong selection bias. Future research should prioritize randomized controlled trials assessing long-term renal function, cost-effectiveness, and potential refinements of robotic vessel sealing technology. A broader, multicenter analysis could provide further insight into optimal device selection for robotic partial nephrectomy.

## Figures and Tables

**Table 1:** Patient demographics and baseline characteristics.

Variable	Vessel Sealer—Group I (n = 54)	Bipolar—Group II (n = 58)	*p*-Value	Test
BMI	28.1	28.9	0.131	*t*-test
Gender (M/F)	45/9	39/19	0.7	Chi-squared
Laterality (R/L)	18/36	26/32	1	Chi-squared
Age	68.5	60.8	0.131	*t*-test

**Table 2:** Comparison of perioperative outcomes between the robotic vessel sealer and bipolar grasper.

Variable	Vessel Sealer—Group I (n = 54)	Bipolar—Group II (n = 58)	*p*-Value	Test
Ischemia Time (min)	24.5 ± 4.81	20.5 ± 12.69	0.44	*t*-test
Blood Loss (mL)	**40** ± **26.83**	**132.5** ± 327.23	**0.037**	*t*-test
Blood Transfusion	1	1	0.159	*t*-test
Operating Time (min)	169.8 ± 11.09	166.9 ± 31.62	0.82	*t*-test
Tumor Diameter by CT (mm)	25.5 ± 14.25	28.38 ± 14.96	0.66	*t*-test
Tumor Diameter by Pathology (mm)	19.6 ± 0.80	23.4 ± 1.19	0.38	*t*-test
Hospital Stay (days)	3 ± 0.00	3.1 ± 1.29	0.84	*t*-test
R.E.N.A.L Score	6.17 ± 1.33	7 ± 1.61	0.2	
Console Time	113.8 ± 13.12	127.9 ± 29.21	0.25	*t*-test
Docking Time	14.7 ± 1.86	18.9 ± 8.80	0.25	*t*-test
Conversion to Open	0	0	1	Chi-squared
Vein Clamping	18	10	1	Chi-squared
Artery Clamping	36	46	0.15	Chi-squared
Artery and Vein Together Clamping	18	10	1	Chi-squared

**Table 3:** Comparison of complications between the robotic vessel sealer and bipolar grasper.

Variable	Vessel Sealer—Group I	Bipolar—Group II	*p*-Value	Test
No	54	58		
Fever	0	0		
Incisional Hernia	0	0		
Deep Vein Thrombosis	0	0		
Ileus	0	0		
Wound Infection	0	0		
Trocar Site Hematoma	0	0		
Hematuria	0	1		
Clavien–Dindo Classification I	0	1		
Clavien–Dindo Classification II–V	0	0		
Overall Complications	0	1	1	Chi-squared

## Data Availability

All the data saved will be provided upon request. The data that support the findings of this study are not publicly available due to patients being from private hospitals, but are available from the corresponding author, M.A.
